# Causal Association between Periodontal Diseases and Cardiovascular Diseases

**DOI:** 10.3390/genes13010013

**Published:** 2021-12-22

**Authors:** Mengchen Zhou, Jiangtao Dong, Lingfeng Zha, Yuhua Liao

**Affiliations:** 1 Department of Cardiology, Union Hospital, Tongji Medical College, Huazhong University of Science and Technology, Wuhan 430022, China; zhmc9990@163.com; 2 Department of Cardiovascular Surgery, Union Hospital, Tongji Medical College, Huazhong University of Science and Technology, Wuhan 430022, China; dongjt0727@foxmail.com; 3 Key Lab of Molecular Biological Targeted Therapies of the Ministry of Education, Union Hospital, Tongji Medical College, Huazhong University of Science and Technology, Wuhan 430022, China

**Keywords:** periodontal diseases, cardiovascular diseases, causal association, Mendelian randomization

## Abstract

Observational studies have revealed that dental diseases such as periodontitis and dental caries increase the risk of cardiovascular diseases (CVDs). However, the causality between periodontal disease (PD) and CVDs is still not clarified. In the present study, two-sample Mendelian randomization (MR) studies were carried out to assess the association between genetic liability for periodontal diseases (dental caries and periodontitis) and major CVDs, including coronary artery disease (CAD), heart failure (HF), atrial fibrillation (AF), and stroke—including ischemic stroke as well as its three main subtypes—based on large-scale genome-wide association studies (GWASs). Our two-sample MR analyses did not provide evidence for dental caries and periodontitis as the causes of cardiovascular diseases; sensitivity analyses, including MR–Egger analysis and weighted median analysis, also supported this result. Gene functional annotation and pathway enrichment analyses indicated the common pathophysiology between cardiovascular diseases and periodontal diseases. The associations from observational studies may be explained by shared risk factors and comorbidities instead of direct consequences. This also suggests that addressing the common risk factors—such as reducing obesity and improving glucose tolerance—could benefit both conditions.

## 1. Introduction

Cardiovascular disease (CVD) is a common and complex disease, especially in middle-aged people those over 50 years old; its morbidity, disability, and mortality rates are relatively high, posing a serious threat to human health [1]. Every year, the number of people dying from CVD is as high as 17 million, ranking first among all causes of death. Ischemic heart disease, stroke, heart failure (HF), cardiomyopathy, and atrial fibrillation (AF) account for more than 95% of cardiovascular-disease-related deaths [1]. CVD is caused by heredity, environment, and their interactions; conventional risk factors for CVD are mainly lifestyle risk factors, including smoking, lipid metabolism disorders, hypertension, and altered glucose metabolism, all of which can be improved by lifestyle improvements. Currently, genetic studies have found a variety of CVD susceptibility genes. As knowledge of cardiovascular disease continues to increase, several chronic infectious, inflammatory, and immune diseases—such as periodontitis—are being found to be related to a significantly increased risk of adverse cardiovascular events.

Periodontitis is one of the most common inflammatory diseases worldwide, with an incidence rate of 20–50% [2]. Periodontitis is common in adults, and is the sixth most prevalent disease globally, characterized by the gradual disintegration of the tooth-supporting apparatus. The World Health Organization reports that periodontitis is the leading cause of tooth loss in adults. Dental caries and periodontitis are prevalent in adults, especially in individuals who are older, leading to momentous health and financial burdens [3]. Epidemiological studies have indicated that severe loss of support structure and tooth loss caused by advanced periodontitis affect ~15% of the world’s population, mainly affecting adults, and whose morbidity increases with age in all populations. Familial and twin studies have emphasized the role of genetics in chronic periodontitis.

There is now substantial evidence supporting an independent association between severe periodontitis and CVDs. Periodontal disease (PD) may lead to an overall burden of inflammation in the body, and can play roles in the pathogenesis of CVDs [4]. Evidence-based studies have revealed that dental diseases—such as periodontitis, dental caries, and tooth loss—increase the risk of diabetes mellitus, atherosclerosis (AS), stroke, coronary artery disease (CAD), HF, and AF [5]. Research has shown that patients with moderate to severe periodontitis have a higher risk of an initial cerebrovascular event than patients without periodontitis or with mild periodontitis [6]; people suffering from periodontitis have more than twice the risk of stroke than periodontally healthy people [7]. Several studies have also reported a positive relationship between periodontitis and HF. A large-scale study from Asia based on the Taiwanese National Health Insurance Research Database suggests that the incidence of AF in patients with periodontal disease is significantly higher than that in patients without periodontal disease [8]. Recently, large-cohort studies have indicated that both new-onset and prevalent periodontitis are related to increased CAD risk, and in patients with stable CAD there are graded associations between tooth loss, stroke, and cardiovascular and all-cause death [9].

However, the causality between PDs and CVDs remains unclear, since observational studies are impeded by reverse causal bias and residual confusion. Due to the nature of the study design, the existence of potentially significant confounding effects may bias the results of observational studies. Mendelian randomization (MR) is a kind of data analysis method that is mainly used in epidemiological etiology inference [10]. Alleles are randomly assigned to progeny gametes during gamete formation based on the Mendelian independent distribution law. Therefore, the associations between genes and diseases are not influenced by common confounding factors such as postnatal environment, socioeconomic status, or behavioral factors, and the causal timing is reasonable, making the effect estimates closer to the real situation.

Here, we performed an MR study that relied on the genome-wide association studies (GWASs) of large samples to investigate the exact causal relationship between PDs and CVDs.

## 2. Materials and Methods

### 2.1. Study Design

In the present study, two-sample MR was carried out to assess the association between genetic liability for periodontal diseases (dental caries and periodontitis) and major CVDs, consisting of CAD, HF, AF, and stroke, including any ischemic stroke (AIS) as well as its three main subtypes: cardioembolic stroke (CES), large-artery atherosclerotic stroke (LAS), and small-vessel stroke (SVS).

### 2.2. Data Sources

This study relied on summarized data from GWASs that are publicly available. Summary data, including the associations between single-nucleotide polymorphism (SNP) and the phenotype of interest, were acquired from the public databases of different consortiums: GWASs summarized data for dental caries and periodontitis from the GLIDE Consortium and UK Biobank (*n* = 487,823), which are the largest GWAS studies to date [11]; CAD from the CARDIoGRAMplusC4D Consortium (*n* = 184,305) [12]; HF from the UK Biobank (*n* = 977,323) [13]; AF from the Atrial Fibrillation Consortium (*n* = 588,190) [14]; and stroke from the MEGASTROKE Consortium (*n* = 521,612) [15]. All GWASs were performed in European ancestry populations, and received relevant ethical approval and participant consent; no additional ethical approval was required.

### 2.3. Mendelian Randomization Analyses

We estimated MR effects using the inverse-variance weighted method (IVW), and performed sensitivity analyses that were more sensitive to the presence of pleiotropic SNPs via the MR–Egger and weighted-median methods. The slope of the MR–Egger regression line can be used to estimate the size of directional pleiotropy.

### 2.4. SNP and Gene Functional Annotation

We used two gene mapping methods to identify genes related to the 47 SNPs associated with dental caries and periodontitis: The first method was based on where the SNP is located in the genome from HaploReg (version 4.1, http://pubs.broadinstitute.org/mammals/haploreg/haploreg_v4.php, accessed on 10 August 2021)), developed for researchers to discover the mechanistic hypotheses of the effects of SNPs on clinical phenotypes; in this method, we mapped 66 genes. The second method was based on eQTL from GTEx (https://www.gtexportal.org/home/index.html, accessed on 10 August 2021), which annotates the effects of SNPs on expression genes; in this method, we mapped 68 genes. Thirteen of these genes were present in both methods.

### 2.5. Functional and Pathway Enrichment Analyses

Various enrichment analyses, including GO (http://geneontology.org, accessed on 24 September 2021) analysis and KEGG (https://www.kegg.jp, accessed on 27 September 2021) analysis, were conducted using Functional Enrichment Analysis Tool—an independent software tool for gene and protein functional enrichment and interaction network analysis (FunRich, version 3.1.3)—and A Gene Annotation & Analysis Resource (Metascape, version 3.5, https://metascape.org, accessed on 28 September 2021), including biological pathway enrichment analyses, protein interaction network structure analyses, and rich gene annotation functions.

### 2.6. Statistical Analyses

Considering the problem of multiple tests, 2-sided *p*-values < 0.006 (=0.05/8 outcomes) were considered to be statistically significant.

## 3. Results

### 3.1. Instrumental Variables

Forty-seven independent SNPs associated with dental caries and periodontitis identified by the latest GWAS (*p* < 5 × 10^–8^) were selected for analysis [11] (Table 1). We excluded SNPs that were absent and/or significant in GWASs of CVDs; among them, 44 SNPs were incorporated into the analyses of CAD and HF, 36 SNPs in the analyses of AF, and 41 SNPs in the analyses of stroke (42 SNPs in ischemic stroke, 43 SNPs in cardioembolic stroke and large-artery atherosclerotic stroke, and 37 SNPs in small-vessel stroke). According to the allelic direction in different GWASs, the effects of SNPs were adjusted to the same direction in each GWAS.

### 3.2. Causal Associations between Periodontal Disease and CVDs

**Figure 1:**
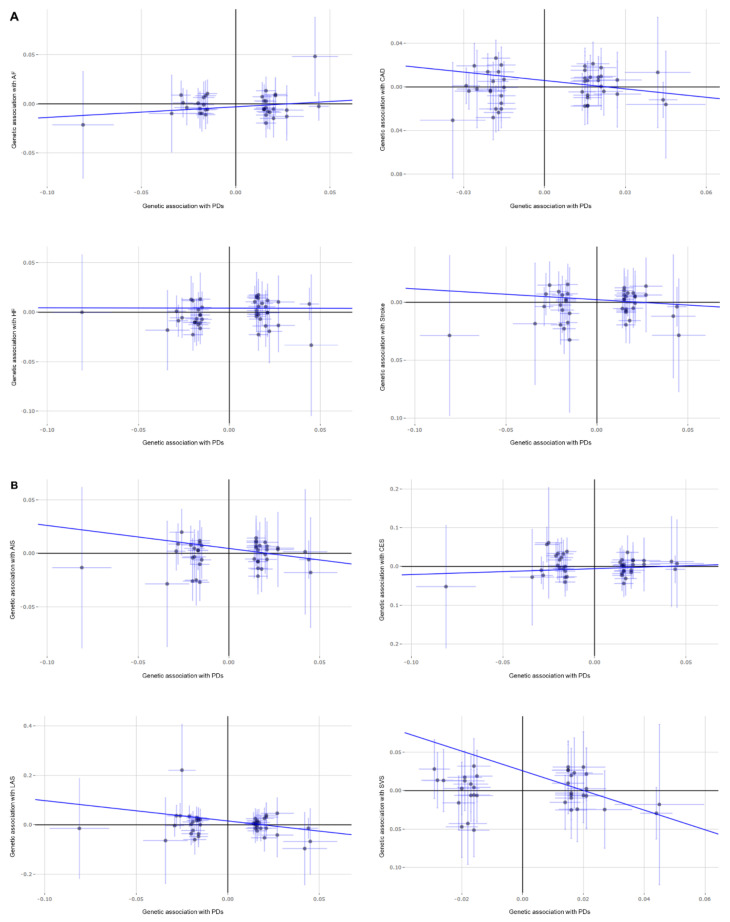
MR analysis for the association of genetically proxied dental caries and periodontitis with cardiovascular diseases: (**A**) MR analysis for CAD, HF, AF, and stroke; (**B**) MR analysis for any ischemic stroke (AIS) as well as its three main subtypes (CES, LAS, and SVS).

The results in the standard MR analyses (IVW) showed no causal correlation between dental caries/periodontitis and CAD, AF, stroke, or AIS and its three main subtypes (*p* > 0.05). A causal relationship may exist between dental caries, periodontitis, and HF (*p* = 0.015) but, unfortunately, it did not pass the multiple tests for statistical significance (*p* > 0.006). Through the advantages of MR analyses, we found that there were no causal relationships between dental caries, periodontitis, and major CVDs (Figure 1).

### 3.3. Sensitivity Analyses

First, we carried out the WM method for sensitivity analysis. Results from this method suggested no causal correlation between dental caries/periodontitis and CAD, AF, HF, stroke, or AIS and its three main subtypes (*p* > 0.05); these results are consistent with those of the IVW method.

**Figure 2:**
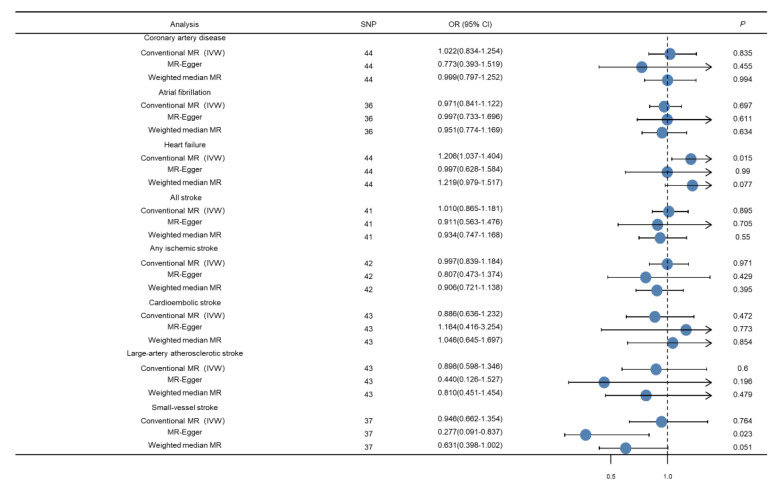
Forest plot depicting MR results for the association of genetically proxied dental caries and periodontitis with cardiovascular diseases.

Next, we conducted MR–Egger analysis; almost all intercept terms were near the origin, thus suggesting that there was no horizontal pleiotropy. The MR–Egger analysis suggested a potential negative causality between dental caries, periodontitis, and SVS (*p* = 0.023); this was not consistent with the results of IVW and WM; however, it did not pass the multiple tests for statistical significance (*p* > 0.006). No other significant causal associations were identified in this MR–Egger analysis. The sensitive analysis supports our previous estimations of the MR; the causal relationships are enhanced by the consistency of results between different methods (Figure 2).

### 3.4. SNP and Gene Functional Annotation

**Figure 3:**
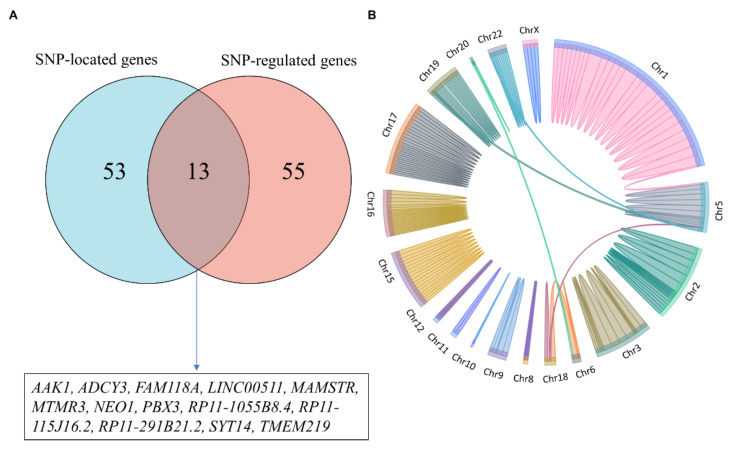
Genes associated with dental caries and periodontitis: (**A**) Genes related to the 47 SNPs; (**B**) SNPs and genes located in the genome.

Prediction of target genes and possible mechanisms of GWAS-related SNPs via systematic data mining is an essential step in interpreting GWAS signal functions. By integrating multiple databases and carrying out a series of functional annotations for dental-caries- and periodontitis-related SNPs, we found that 47 SNPs were located in 66 gene regions, and could affect the expression of 68 genes through cis-eQTL and/or trans-eQTL (Figure 3); among them, only 13 genes (*AAK1*, *ADCY3*, *FAM118A*, *LINC00511*, *MAMSTR*, *MTMR3*, *NEO1*, *PBX3*, *RP11-1055B8.4*, *RP11-115J16.2*, *RP11-291B21.2*, *SYT14*, and *TMEM219*) were both SNP-located and regulated by these SNPs, which may play a central role in the pathological process of disease (Figure 3).

### 3.5. Pathway and Functional Enrichment Analyses

**Figure 4:**
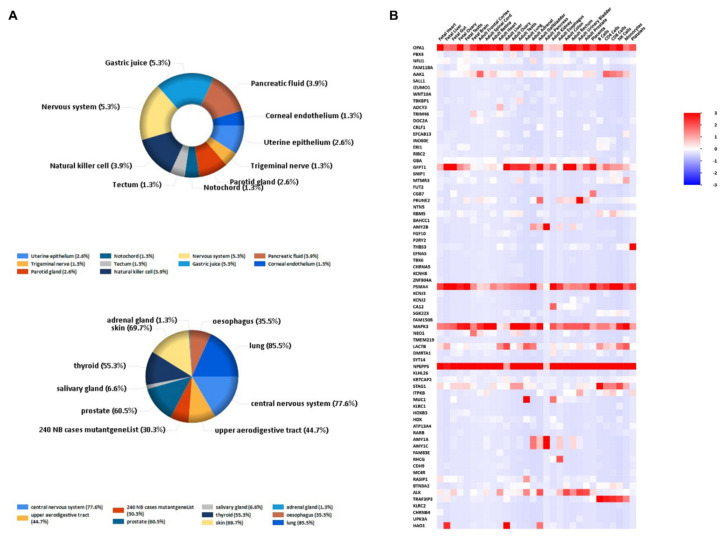
Dental-caries- and periodontitis-related gene expression distribution: (**A**) gene expression in human tissues and systems; (**B**) heatmap of each gene expression distribution.

**Figure 5:**
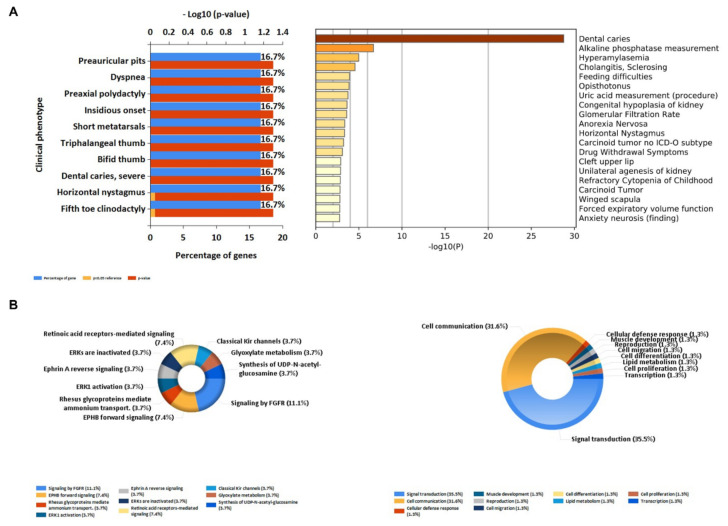
Dental-caries- and periodontitis-related genes’ functional annotation: (**A**) the clinical phenotypes of the related genes; (**B**) the biological pathways and processes of the related genes.

**Figure 6:**
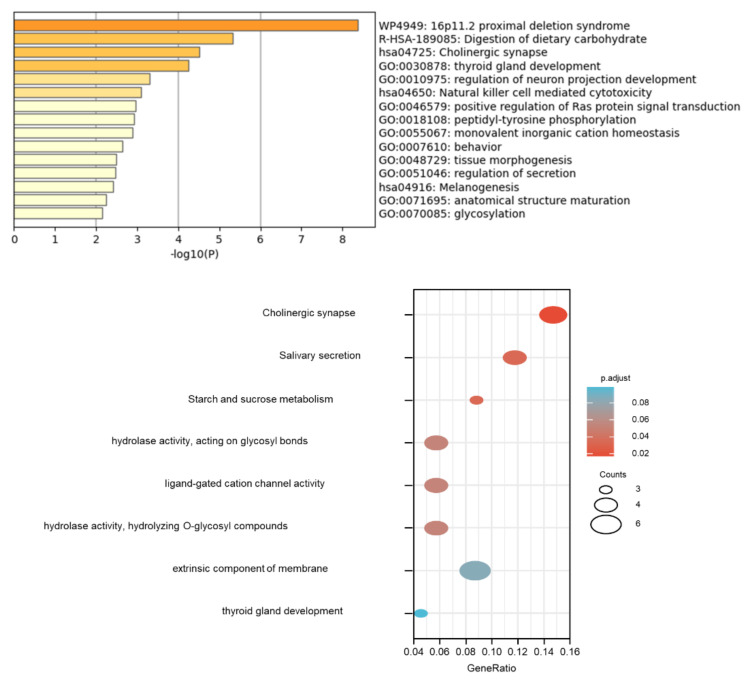
Dental-caries- and periodontitis-related genes in the enrichment of GO and KEGG.

**Figure 7:**
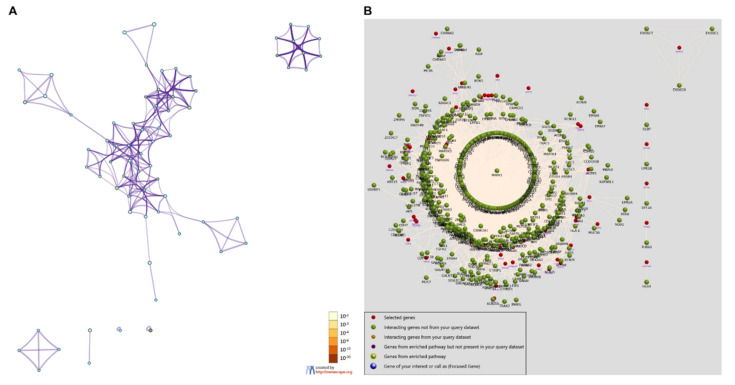
Interactions between genes and a network layout of the KEGG and GO clusters: (**A**) gene clusters; (**B**) network of gene interactions.

We conducted gene cluster analysis for 121 genes annotated by the SNPs above to explore their possible biological functions through various functional enrichment analysis tools. The genes tended to be expressed in the esophagus, lungs, central nervous system, upper aerodigestive tract, gastric juice, and pancreatic fluid. Several genes—such as *OPA1*, *ADCY3*, *PSMA4*, *LACTB*, *NPEPPS*, and *ALK*—are highly expressed in the adult heart (Figure 4); among them, *OPA1*-related pathways are glucose/energy metabolism, while *ADCY3* takes part in regulating insulin levels and body fat amassing in response to a high-fat diet, and the related disease is a body mass index quantitative trait. The pathways related to *NPEPPS* are Class I MHC-mediated antigen processing and presentation, as well as the innate immune system, while *ALK* is involved in the resistance to weight gain. We used DisGeNET (http://www.disgenet.org, accessed on 28 September 2021), which is a comprehensive database for integrating information about genes and variants associated with human diseases, to analyze the clinical phenotypes related to these genes, and found that they were mainly related to dental caries and hyperamylasemia (Figure 5). The biological processes involved in these genes are signal transduction, cell communication and migration, muscle development, and lipid metabolism; the biological pathways involved in these genes are glyoxylate metabolism, signaling by FGFR, ERK1 activation, and retinoic-acid-receptor-mediated signaling (Figure 5). KEGG and GO enrichment pathway analyses indicated that those genes were significantly enriched in glycosylation, the digestion of dietary carbohydrate, cholinergic synapse, salivary secretion, and starch and sucrose metabolism, as well as the ligand-gated cation channel activity pathway (Figure 6). There were complex interactions between those genes, and a network layout was shown by the KEGG and GO clusters (Figure 7).

## 4. Discussion

**Figure 8:**
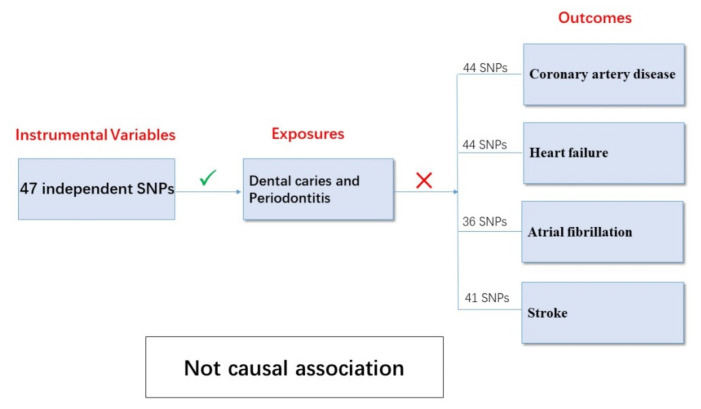
Association between dental caries/periodontitis and cardiovascular diseases.

The relationship between PDs and CVDs has potentially significant public health implications due to their high prevalence. A prospective study from the National Health Insurance System—National Health Screening Cohort (NHISHEALS), including 247,696 individuals without any history of CVD, reported that the presence of periodontitis and the increasing number of dental caries lesions, as well as the increasing number of teeth lost, were all related to an increased risk of future major cardiovascular events (MACEs), including AMI, HF, stroke, and cardiovascular death [16]. The available evidence has shown that PD is related to CVDs, independent of known confounding factors; however, this evidence is mainly from investigative studies and, therefore, does not prove that PD is the cause of CVDs, or that periodontal therapy can prevent CVDs or change their clinical process. Our two-sample MR analyses did not provide evidence for dental caries and periodontitis as the causes of CVDs (Figure 8). Our results are consistent with the recently published study by Steven et al., which also found no causal relationship between periodontitis and ischemic stroke or CAD; however, only five SNPs previously reported to be associated with periodontitis were included in their analysis [17]. For this study, 47 independent SNPs associated with dental caries and periodontitis, identified by the most recent and the hitherto largest GWAS, were selected for analysis [11]. Furthermore, considering that clinical studies have also observed a close relationship between periodontitis and other CVDs, our study also analyzed the causal relationship between dental caries/periodontitis and AF/HF. Our results not only strengthen the evidence of the relationship between periodontitis and ischemic stroke and CAD, but also add to the evidence of the causal relationship between periodontitis and other major cardiovascular diseases. We systematically analyzed the causal relationship between periodontitis and major CVDs, but the results of this causal study are inconsistent with those of observational studies. The associations from observational studies may be explained by shared risk factors and comorbidities, rather than being a direct consequence.

Various mechanisms have been suggested for the relationship between PDs and CVDs, including bacteremia and associated systemic inflammatory sequelae [18,19,20]. Periodontal diseases can cause inflammation and immune responses, both locally and systematically (increasing white blood cell count, CRP, fibrinogens, cell adhesion molecules, and pro-inflammatory cytokines) [21]. Systemic inflammation and/or immune response to periodontal infection could raise the risk of CVDs. Pathogens from the mouth can enter atherosclerotic plaques through the bloodstream, promoting inflammation or immune responses within atherosclerotic plaques. Multiple oral bacterial pathogens and bacterial DNAs have been discovered in atherosclerotic plaques. In animal studies, Porphyromonas gingivalis (P. gingivalis) infection increases atherosclerotic plaque volume and accumulates cholesterol esters and inflammatory mediators. Moreover, compared with the control group, serum IgA antibodies to P. gingivalis are higher in patients with MI.

A growing number of patients and providers claim that PD treatment strategies protect against CVD. The Atherosclerosis Risk in Communities (ARIC) study, including 6736 individuals after 15 years of follow-up, reported that self-reported regular dental care individuals had a lower risk of ischemic stroke (HR = 0.77) than the episodic care individuals [6]. Brushing more than once a day was related to a lower incidence of atherosclerotic CVD events (HR = 0.91), while regular professional cleaning further lowered the risks (HR = 0.86) [16]. Moderate evidence indicates that periodontal treatment reduces systemic inflammation by reducing CRP, and also reduces the incidence of CVD events [5,16]. Several intervention trials have suggested that periodontal treatment via a variety of methods—such as intensive mechanotherapy, antimicrobial therapy, or extraction—improve endothelial function and reduce biomarkers of atherosclerotic vascular disease (ASVD) (IL-6, CRP)—especially in individuals already suffering from ASVD [22,23]. Available data suggest an overall trend of periodontal-treatment-induced systemic inflammatory suppression and improvement of noninvasive markers of ASVD and endothelial function.

While the forceful pathophysiological basis highlights the importance of these mechanisms, the relationship between periodontitis and CVDs may not be causal. Cardiovascular and periodontal diseases share several risk factors—for instance, age, smoking, diabetes, and poor socioeconomic conditions. In nearly all of the observational research, at least some of the associations could be illuminated by adjusting for cardiovascular risk factors [24]. Evidence of a variety of common risk factors is related to understanding of the likely common pathophysiology of CVDs and PDs. Studies have also shown benefits for both diseases from not smoking, reducing obesity, improving glucose tolerance, and addressing other common risk factors [4]. A number of inflammatory and oxidative stress markers are shared by CVDs and periodontitis; therefore, the possibility that periodontitis may affect CVDs is premised on elevated levels of serum inflammatory markers in patients with periodontitis compared to healthy individuals with periodontitis or patients treated for periodontitis. Studies have also found that serum lipid levels—such as total cholesterol, low-density lipoprotein, triglyceride, very-low-density lipoprotein, and oxidized low-density lipoprotein—are ascended in periodontitis, while high-density lipoprotein (HDL) levels are lower in periodontitis compared with controls; however, these levels are reversed after periodontal treatment [25]. In addition, as is well known, lipid-lowering therapy by statins reduces the cardiovascular event risk, but statins also play a positive role in periodontal health; recent studies have indicated the beneficial effects of statins on periodontal status [26,27]. There is finite evidence that statin may reduce periodontal inflammation by reducing cholesterol deposits near the periosteum, which may augment the inflammatory responses [26,28,29]. Both periodontitis and cardiovascular disease are hereditary diseases with many susceptibilities and pathogenic genes. The recognition that genes associated with chromosome 9p21, as well as genes associated with the regulation of transforming growth factors, are susceptible to periodontitis as well as CAD provides further evidence that common pathophysiological pathways play important roles in both diseases [30]. In this study, through functional annotation and gene pathway aggregation analysis, we found that genes associated with periodontitis were also highly expressed in the heart; among them, *OPA1*-related pathways concern glucose/energy metabolism; *ADCY3* takes part in regulating insulin levels and body fat amassing in response to a high-fat diet, and the related disease is the body mass index quantitative trait; the pathways related to *NPEPPS* cover Class I MHC-mediated antigen processing and presentation, as well as the innate immune system; and *ALK* is involved in resistance to weight gain. These genes may still influence the occurrence and progression of both diseases through common risk factors. In addition, considering the effect of SNP pleiotropy, we tried to exclude those SNPs with a significant tendency in GWASs of CVDs when exploring the relationship between periodontitis and CVDs. These pleiotropic genetic loci may be the common genetic basis of both diseases. Currently, the relationship between periodontitis and CVDs and the specific mechanism between them are subject to discussion, and many studies focus on the impact of periodontal therapy on CVDs, but the focus is still on inflammation and anti-inflammatory treatments. Today, there is also awareness of the cardiovascular risks and complications associated with periodontal interventions, as well as the pathogenesis- and treatment-related effects of statins on periodontitis [5]. With cardiovascular and dental experts working together, more related research and results are expected.

As reported, severe periodontitis is not only associated with cardiovascular mortality, but is also significantly associated with all-cause mortality [31]. Periodontal health and oral hygiene are not only dentists’ concerns, but also our own responsibility. Although there is no causal relationship between periodontitis and CVD, periodontitis may still be a modifiable, nontraditional risk factor for CVD. The effect of periodontal therapy is associated with a significant reduction in levels of CRP, which is an inflammatory marker [23]. Observational studies have found that any oral health intervention—including improved oral hygiene practices, periodontal treatment, and dental prophylaxis—can reduce the incidence of CVD events [16,32,33]. People diagnosed with CVDs should receive a full oral examination, while dental patients should pay more attention to CVD risk factors such as diabetes, obesity, smoking, hypertension, hyperlipidemia, and hyperglycemia, as they can also lead to periodontal disease. In other words, patients with periodontitis and cardiovascular disease are advised to regularly adhere to dental treatment, maintenance, and prevention, as well as to control the commonly shared risk factors; meanwhile, it is also important for everyone to have regular oral and dental care in order to maintain oral health.

Our study has strengths. First, compared to previous studies, the causal relationship between dental caries, periodontitis, and CVDs was more systematically evaluated. Second, the MR study reduced the bias of conventional confounders and reverse causality compared with observational studies, and the reliability of the results was enhanced by using three MR analysis methods. Third, a large sample of GWAS data, taken from the latest as well as the hitherto largest GWASs, was used to ensure strong statistical power. Finally, all samples analyzed were from European populations, so as to avoid bias due to population stratification.

Our study also has limitations. First, the data in our analysis are mainly from European ancestry, with a lack of data for other populations, leading to our findings being less generalizable to other ancestries. Second, despite our best efforts to lessen the effect of pleiotropic bias, a few SNPs still showed pleiotropy, which may affect the accuracy of MR assessment. Third, partial individuals are overlapped in the GWASs of HF and AF, which could cause a slight bias in the MR estimations of HF and AF, thus affecting the reliability and accuracy of the MR results; however, the actual overlap is limited, and does not affect our conclusions. Finally, we only discussed the causal relationship between dental caries, periodontitis, and CVDs from a genetic perspective; the specific mechanisms between them still require further experiments for confirmation.

## 5. Conclusions

In conclusion, the present two-sample MR analyses did not provide evidence for dental caries and periodontitis as the causes of cardiovascular diseases. Investigating the precise shared risk factors will contribute to controlling the incidence of cardiovascular diseases, thus improving public health.

## Figures and Tables

**Table 1:** Forty-seven independent SNPs associated with dental caries and periodontitis.

No	Gene	SNP	EA: EAF	β (SE)	*p*-Value
1	*AMY1C*	rs72694438	a:0.21	0.021(0.0033)	2.25 × 10^−10^
2	*KRTCAP2*	rs4971099	a:0.55	−0.021(0.0027)	7.47 × 10^−15^
3	*SYT14*	rs2046850	t:0.19	−0.019(0.0033)	1.77 × 10^−8^
4	*ITPKB*	rs3820640	t:0.84	0.020(0.0037)	2.65 × 10^−8^
5	*KCNJ3*	rs2652452	a:0.45	−0.016(0.0027)	3.27 × 10^−9^
6	*ZNF804A*	rs263771	a:0.23	0.018(0.0031)	6.53 × 10^−9^
7	*WNT10A*	rs121908120	a:0.03	−0.081(0.0083)	2.03 × 10^−22^
8	*ADCY3*	rs11676272	a:0.52	−0.015(0.0026)	1.07 × 10^−8^
9	*ALK*	rs80270335	t:0.09	0.027(0.0045)	2.1 × 10^−9^
10	*FAM150B*	rs62106258	t:0.95	0.042(0.0062)	8.6 × 10^−12^
11	*AAK1*	rs5831974	d:0.45	−0.016(0.0027)	6.97 × 10^−10^
12	*STAG1*	rs61790808	a:0.64	−0.016(0.0028)	9.1 × 10^−9^
13	*KCNH8*	rs9831002	t:0.49	−0.016(0.0026)	8.79 × 10^−10^
14	*OPA1*	rs185566659	a:0.032	0.045(0.0075)	1.54 × 10^−9^
15	*RARB*	rs7429279	a:0.41	0.016(0.0027)	1.28 × 10^−9^
16	*RBM5*	3:50135699DI	d:0.48	−0.018(0.0027)	4.79 × 10^−12^
17	*EFNA5*	rs1352724	a:0.22	−0.018(0.0032)	3.64 × 10^−8^
18	*C5orf66*	rs1122171	t:0.59	0.044(0.0027)	2.84 × 10^−62^
19	*CDH9*	rs55769264	a:0.45	0.015(0.0027)	2.84 × 10^−8^
20	*FGF10*	rs1482698	c:0.38	0.020(0.0027)	1.47 × 10^−13^
21	*HLA*	rs9366651	t:0.51	−0.029(0.0026)	2.66 × 10^−28^
22	*LOC157273*	rs898797	t:0.59	0.015(0.0027)	1.52 × 10^−8^
23	*PBX3*	rs10987008	a:0.64	0.021(0.0028)	7.47 × 10^−14^
24	*DMRTA1*	rs10811723	a:0.30	−0.019(0.0029)	3.41 × 10^−11^
25	*PRUNE2*	rs7852129	a:0.89	−0.025(0.0038)	7.91 × 10^−11^
26	*STFA1P*	rs7918807	t:0.52	0.015(0.0027)	3.58 × 10^−8^
27	*P2RY2*	rs149467613	a:0.05	−0.034(0.0062)	3.21 × 10^−8^
28	*KLRAP1*	rs10772314	a:0.40	−0.015(0.0027)	3.16 × 10^−8^
29	*CA12*	rs72748935	t:0.46	−0.028(0.0027)	1.31 × 10^−26^
30	*NEO1*	rs6495046	c:0.36	−0.017(0.0028)	3.47 × 10^−10^
31	*CHRNA3*	rs10851907	a:0.41	0.016(0.0027)	1.03 × 10^−9^
32	*RHCG*	rs2072693	t:0.48	0.014(0.0026)	4.92 × 10^−8^
33	*TMEM219*	rs8054556	a:0.46	0.016(0.0027)	2.23 × 10^−9^
34	*SALL1*	rs1108343	t:0.36	0.016(0.0028)	1.32 × 10^−8^
35	*FOXL1*	rs10048146	a:0.81	−0.026(0.0034)	5.2 × 10^−14^
36	*NPEPPS*	rs3865314	a:0.51	0.015(0.0026)	1.48 × 10^−8^
37	*HOXB-AS2*	rs9905793	a:0.091	0.027(0.0046)	6.51 × 10^−9^
38	*KCNJ2*	rs34559440	t:0.68	−0.016(0.0028)	1.14 × 10^−8^
39	*LOC100499467*	rs7217268	a:0.38	0.016(0.0027)	1.48 × 10^−9^
40	*BAHCC1*	rs57067187	t:0.63	0.015(0.0027)	6.9 × 10^−9^
41	*MC4R*	rs28822480	a:0.29	0.021(0.0029)	7.08 × 10^−13^
42	*CRLF1*	rs2238651	t:0.24	0.017(0.0031)	4.39 × 10^−8^
43	*MAMSTR*	rs11672900	a:0.47	−0.020(0.0027)	4.67 × 10^−14^
44	*HAO1*	rs4816017	a:0.29	−0.017(0.0029)	7.08 × 10^−9^
45	*MTMR3*	rs140357883	d:0.84	0.022(0.0036)	2.33 × 10^−9^
46	*FAM118A*	rs1569414	t:0.73	−0.020(0.0030)	1.19 × 10^−11^
47	*HDX*	rs5922945	t:0.34	−0.016(0.0028)	1.55 × 10^−8^

## Data Availability

All data are available on reasonable request from corresponding author.
